# Stimulation of the Sphenopalatine Ganglion Induces Reperfusion and Blood-Brain Barrier Protection in the Photothrombotic Stroke Model

**DOI:** 10.1371/journal.pone.0039636

**Published:** 2012-06-22

**Authors:** Haviv Levi, Karl Schoknecht, Ofer Prager, Yoash Chassidim, Itai Weissberg, Yonatan Serlin, Alon Friedman

**Affiliations:** 1 Departments of Physiology and Neurobiology, Zlotowski Center for Neuroscience, Ben-Gurion University of the Negev, Beer-Sheva, Israel; 2 Institute of Neurophysiology, Neurocure Research Center, Charité Universitätsmedizin, Berlin, Germany; Biological Research Centre of the Hungarian Academy of Sciences, Hungary

## Abstract

**Purpose:**

The treatment of stroke remains a challenge. Animal studies showing that electrical stimulation of the sphenopalatine ganglion (SPG) exerts beneficial effects in the treatment of stroke have led to the initiation of clinical studies. However, the detailed effects of SPG stimulation on the injured brain are not known.

**Methods:**

The effect of acute SPG stimulation was studied by direct vascular imaging, fluorescent angiography and laser Doppler flowmetry in the sensory motor cortex of the anaesthetized rat. Focal cerebral ischemia was induced by the rose bengal (RB) photothrombosis method. In chronic experiments, SPG stimulation, starting 15 min or 24 h after photothrombosis, was given for 3 h per day on four consecutive days. Structural damage was assessed using histological and immunohistochemical methods. Cortical functions were assessed by quantitative analysis of epidural electro-corticographic (ECoG) activity continuously recorded in behaving animals.

**Results:**

Stimulation induced intensity- and duration-dependent vasodilation and increased cerebral blood flow in both healthy and photothrombotic brains. In SPG-stimulated rats both blood brain-barrier (BBB) opening, pathological brain activity and lesion volume were attenuated compared to untreated stroke animals, with no apparent difference in the glial response surrounding the necrotic lesion.

**Conclusion:**

SPG-stimulation in rats induces vasodilation of cortical arterioles, partial reperfusion of the ischemic lesion, and normalization of brain functions with reduced BBB dysfunction and stroke volume. These findings support the potential therapeutic effect of SPG stimulation in focal cerebral ischemia even when applied 24 h after stroke onset and thus may extend the therapeutic window of currently administered stroke medications.

## Introduction

In cerebral stroke – a leading cause of morbidity and mortality in the aged population – the ischemic brain is typically characterized by a central core with very low perfusion which undergoes necrotic cell death, and a surrounding dysfunctional peri-ischemic region, also often known as the ‘stroke penumbra’ [1]. Neuronal dysfunction in the peri-ischemic region appears to arise from several interrelated processes including reduced energy supply, metabolic and ionic disturbances, vascular compromise, blood-brain barrier (BBB) dysfunction, glial and immunological response [2], [3]. It is the fate of the peri-ischemic region that often determines the extent of the patient’s recovery. Importantly, stroke may progress or become hemorrhagic and the penumbra may become part of the necrotic region. Furthermore, even if the peri-ischemic region does escape cell death, recovery may not be complete and this zone may become dysfunctional, epileptic or undergo delayed neurodegeneration.

To this end, available treatments in stroke focus on improving brain tissue perfusion. Targeting vascular injury/BBB dysfunction or immune response within the peri-ischemic region is not yet a treatment option (however see [4]). Thus, intravenous recombinant tissue plasminogen activator (IV r-tPA) and mechanical recanalization remain the only approved treatments for stroke. The fundamental limitation of these therapies is the short treatment window (4–5 h from stroke onset for IV r-tPA and <8 h for the use of the recanalization devices [5]). Moreover, symptomatic hemorrhagic transformation of the infarct associated with the administration of IV r-tPA remains a primary concern [6]. In the USA, for example, only about 4% of ischemic stroke patients are treated with IV r-tPA [7]. Hence, there is substantial interest in developing novel therapeutics for the treatment of acute cerebral ischemia.

A possible treatment route that is currently being studied is stimulation of the sphenopalatine ganglion (SPG) ((http://clinicaltrials.gov/ct2/show/NCT00826059?term=Brainsgate&rank=1), [8]). The SPG is the source of parasympathetic innervations to the anterior cerebral circulation, which comprises the middle cerebral artery, the anterior cerebral artery, and their tributaries. Studies in rodents, dogs and monkeys have demonstrated that stimulating neurons within the SPG after permanent middle cerebral artery occlusion (MCAO) leads to an ipsilateral increase in regional cerebral blood flow (rCBF) [9]–[13] and, at high stimulation intensities, to BBB breakdown [14]. In a magnetic resonance imaging (MRI) study in rats, Henninger and Fisher (2007) [15] reported that a single train (four sets) of SPG stimulation, 15 minutes after MCAO led to elevated rCBF in the peri-ischemic region and to a significantly reduced infarct volume 24 h after treatment. In accordance, a recent study has shown that resection of the SPG leads to an increased infarct volume following MCAO [16].

The aims of the present study were to use imaging methods to test directly the effect of SPG stimulation on vascular diameter and permeability, rCBF, infarct size and neuronal network activity in the acutely ischemic cortex. A better insight into the mechanisms underlying the effects of SPG may improve patient selection and follow-up, and improve the use of SPG stimulation as a new therapeutic modality for brain disorders.

## Materials and Methods

All experimental procedures were approved by the Animal Ethics Committee of Ben-Gurion University of the Negev, Beer-Sheva, Israel. Chemicals were purchased from Sigma-Aldrich unless otherwise stated. All images were processed by “in house” developed scripts (MATLAB 2009b).

### Surgical Procedure

Adult male Sprague-Dawley rats, each weighing 300–400 g, were deeply anesthetized by intraperitoneal injection of thiopental (6 mg/100 g) for the acute experiments and ketamine (100 mg/ml, 0.08 ml/100 g) and xylazine (20 mg/ml, 0.06 ml/100 g) for the chronic experiments. In the acute experiments animals were tracheostomized and ventilated mechanically (SAR-830 Ventilator), and the tail artery and vein were catheterized. Body temperature was continuously monitored and maintained at 37±0.5°C by using a heating pad. Craniotomy was performed as previously described [17] (1 mm caudal and 1 mm lateral to bregma; bone window dimensions 2×5 mm). The dura was removed, and the exposed cortical surface was superfused with oxygenated (95% O_2_, 5% CO_2_) artificial cerebrospinal fluid (ACSF) containing (in mM) 129 NaCl, 21 NaHCO_3_, 1.25 NaH_2_PO_4_, 1.8 MgSO_4_, 1.6 CaCl_2_, 3 KCl, and 10 glucose (pH 7.4).

### Positioning of Stimulation Electrode

Following craniotomy, a fine bipolar hook stimulating electrode (NeuroPath, BrainsGate) was implanted in nerve bundles, under a surgical microscope (Zeiss OMPI 9-FC), as described previously [9]. Briefly, scalp incision was made near the right upper orbit. Intra-orbital structures were retracted laterally; nerve bundles, including the nasociliary nerve and parasympathetic nerve fibers, were separated from the SPG and a fine bipolar electrode was placed underneath the bundle.

### Induction of Stroke

Intravascular thrombosis was induced by using the photosensitive dye Rose bengal (RB) [18]. Following intravenous administration of RB (7.5 mg/ml, in saline; 1.33 ml/kg body weight), the brain was exposed to focused monochromatic laser light (561 nm, Optoelectronic Tech lb-WL206) or halogen light (see below) for 15 min.

### Stimulation Protocol for the Acute Experiments

In these experiments, following the surgical procedure, the rat was placed under a stereomicroscope and direct imaging of pial vessels was performed with an EMCCD camera (Andor Technology, DL-658M-TIL). The SPG was stimulated using a previously optimized protocol [13]: 12 stimulation trains were administered; each train was composed of two sets of 60-s stimuli (at 10 Hz) separated by a 12-s interval, followed by 13.6 min of “OFF” time, giving a total treatment time of 180 min. Three protocols comprising different stimulation intensities and durations were tested: a 200-µs pulse at 1 mA (n = 7), a 200-µs pulse at 2 mA (n = 7), and a 500-µs pulse at 2 mA (n = 7). A control group (n = 6) was exposed to the same treatments but stimulation was set at 0 mA. The most efficient pulse width determined in acute experiments was later used for treatment in the chronic experiments.

### Stimulation Protocol for the Chronic Experiments

The effect of daily SPG stimulation on the long-term outcome after photothrombosis was evaluated by measuring BBB permeability, cortical electrical activity, lesion size and glial response. Surgical procedures were performed as described above. Following RB injection, the cortex was exposed to halogen light. For chronic SPG stimulation, wireless, radio-frequency-activated electrodes were implanted. After induction of photothrombosis, the skin was sutured and the animals were placed in individual cages, where they were kept for 6–21 days. Four treatment groups were studied: (1) photothrombosis treatment without SPG stimulation (designated RB); (2) photothrombosis with SPG stimulation starting 15 min after treatment (RB-SPG-15 min); (3) photothrombosis with SPG stimulation starting 24 h after treatment (RB-SPG-24 h); and (4) control group, not exposed to photothrombosis or stimulation (Sham). In the treated groups rats were stimulated for four consecutive days, 3 h per day. Pulse duration was set to 500 µs. Stimulation intensity was increased gradually from 0.5 mA to a maximum of 2 mA (in 0.1-mA steps) and adjusted individually for each animal until animal showed a behavioral response (e.g. muscle twitching, startle response) to the stimulus and then immediately reduced to a sub-clinical (asymptomatic) level.

### Assessment of Vascular Diameter and rCBF

For measuring vessel diameters, images were taken continuously every 2 s, starting 1 min before stimulation. A segmentation process using noise filtration, hole filling and an adaptive threshold produced a binary image of the blood vessels. Within a vessel of interest, five points were chosen manually, and an average diameter was calculated on the basis of the number of pixels across the vessel. Results were expressed as percent change of baseline diameter.

To follow changes in rCBF, a laser-Doppler probe (Optronix, OxyFlo™ 2000) was mounted on an electrode manipulator and placed 0.2–0.3 mm above the cortical surface [19]. rCBF measurements were taken continuously, with flow values being expressed in arbitrary units. In all experiments, baseline recordings were performed for at least 15 min prior to stimulation, and results were expressed as percent change relative to the baseline. In addition, fluorescent angiography was used to estimate changes in rCBF, as previously reported [20]. Briefly, images (30/s) were taken following a peripheral IV injection of the fluorescent tracer Lucifer yellow CH dipotassium salt (LY, FW = 521.58). Injections were repeated before (30 min) and during (10 min after initiation) stimulation. Regions of interest (ROIs) in arteries and veins were chosen manually and the intensity in each ROI was plotted as a function of time. The peak-to-peak interval from arterial to nearby vein and the average slope of rise (slope to max, incline) were calculated.

### Recording and Analysis of Brain Activity

Electrocorticography was performed using a telemetric system (Data Science International, St. Paul, MN, USA). Electrodes (Transoma Medical) were implanted through newly drilled holes [21] in the epidural space as close as possible to the treated region and held in place with bone-cement. The radio-telemetry pack was inserted into a subcutaneous pocket dorsal to the scapula. ECoG was recorded daily for 3 h before and after SPG stimulation at a sampling rate of 1 kHz.

Recorded ECoG data was analyzed offline: Data was filtered between 2–90 Hz using zero-phase forward and reverse digital band pass filtering (filtfilt, MATLAB). The frequency spectrum was generated using Fourier transform analysis and normalized either for each recording session or to the first recording day of that particular animal. The sum of the power was calculated for the following frequency bands: 2–4, 5–10, 11-45, 46–70 and 71–90 Hz. High amplitude (>3 SD from baseline on day 1) and fast activity events (71–90 Hz) were defined as “seizure like events” and counted.

### Morphology and Immunohistochemistry

BBB opening was evaluated four days after photothrombosis. The albumin-binding dye Evans blue (EB; 1 ml of a 2% solution) was injected into the tail veins of anesthetized animals. Thirty minutes later, animals were perfused transcardially with 4% paraformaldehyde (PFA, 100 ml), and their brains were removed [17]. Images of the cortex of each animal were acquired at low magnification (×6.4) using a CCD camera (AxioCam MRc5) under similar image acquisition settings. BBB opening was evaluated by calculating the average intensity of the blue channel in the treated hemisphere. The brain area with BBB opening was calculated as the percentage of blue pixels in the treated hemisphere vs. a fixed threshold. For morphological studies, which were conducted 14–21 days after the induction of photothrombosis, animals were anaesthetized and perfused with PFA; brains were removed and stored for 48 h in PFA. Coronal sections (40 µm thick, n = 6–8) from the center of the lesion were sliced and stained with cresyl violet (1%, 2 min) for lesion volume measurements. Images were obtained at low magnification (×6.4, Zeiss, AxioCam MRc5). The area of the remaining cortical tissue was measured and the loss of cortical gray matter was assessed on the basis of the number of ipsilateral pixels representing the neocortex in comparison with the contralateral (control) hemisphere.

The glial inflammatory response was evaluated by using immunostaining against the astrocytic marker glial fibrillary acidic protein (GFAP) and the microglial marker ionized calcium binding adapter molecule 1 (Iba1), as previously reported [22]. Slices of 40 µm were treated with 10% normal goat serum for 30 min and incubated overnight at 4°C with polyclonal rabbit anti-GFAP (1∶250, Dako Germany) or polyclonal mouse anti-Iba1 (1∶250, Wako Pure Chemical Industries, Ltd.). For secondary antibodies, Cy5 goat anti-rabbit IgG (1∶100) and Alexa Fluor 555 goat anti-mouse IgG (1∶100, both Invitrogen Corporation) were used. Incubation was conducted for 5 h at room temperature. Images were acquired at 400-fold magnification (20–30 images per section, 5–8 sections per animal) using a confocal laser-scanning microscope (Leica TCS SP2).

### Statistics

All values were expressed as means ±SEM. Statistical analysis for differences between means comparing groups was performed with the non-parametric *Mann-Whitney* Test. p values of <0.05 were taken as significant.

## Results

### SPG Stimulation Leads to Vasodilation and Increased rCBF in the Healthy and Injured Brains

**Figure 1:**
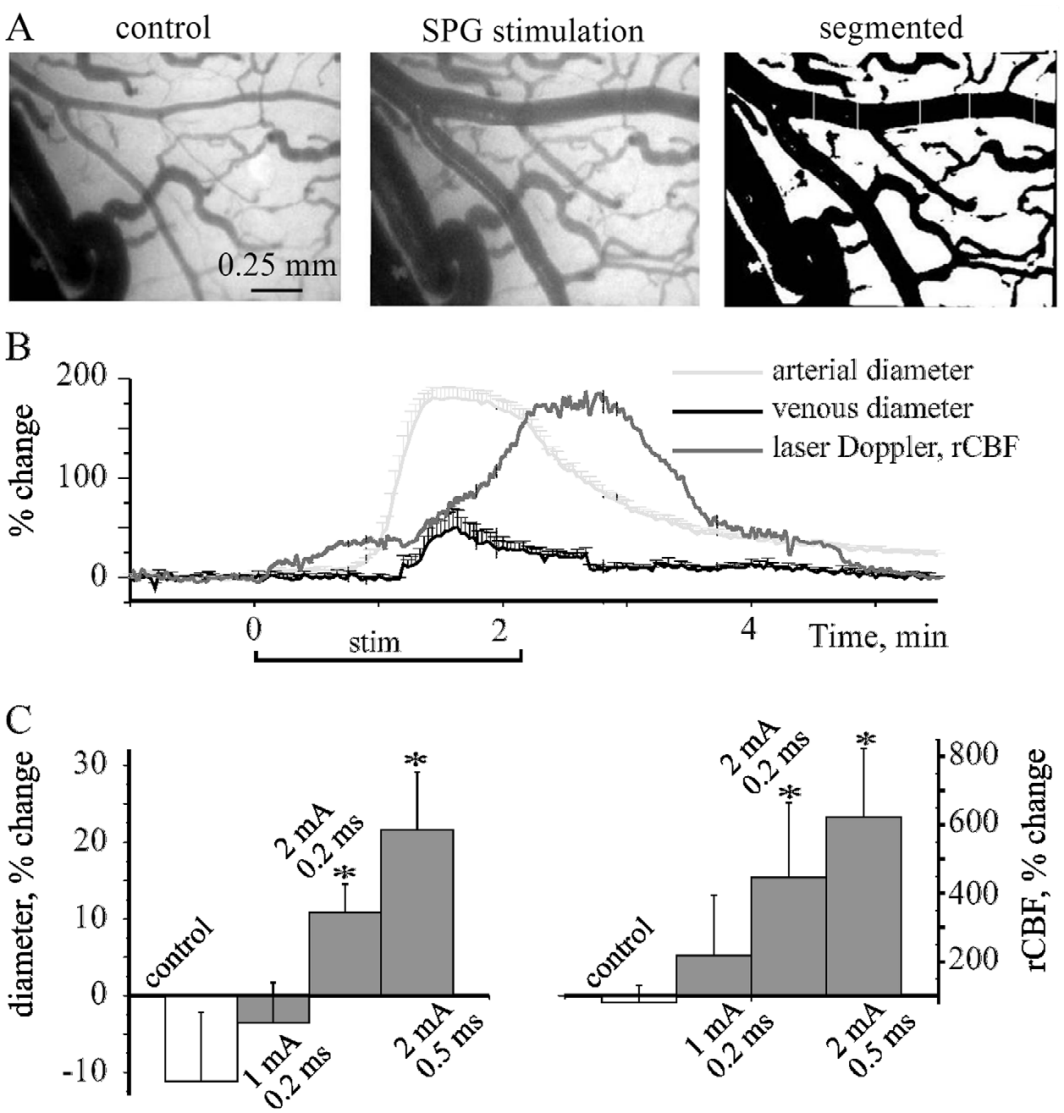
SPG stimulation induces vasodilation in the healthy brain A, Left and middle, respectively, representative image of the brain surface under control conditions and during SPG stimulation: right, the same image after processing for diameter measurement. B, Arterial (red) and venous (blue) changes in diameter (presented as % change from baseline) are shown before, during and after a single stimulation train for the experiment shown in (A). Note that increased rCBF (gray) is following the arterial vasodilation. C, Average % change in arterial diameter (left graph) and rCBF (right graph) during different stimulation protocols (see text). *p<0.05.

We first performed “acute experiments” to test the vascular response to SPG stimulation. Stimulation was associated with a slowly progressing significant dilation of pial vessels. Vasodilatation was first observed in arterioles, followed by a milder dilatation of nearby venules and was associated with increased rCBF (Fig. 1A-B). Notably, vasodilation persisted 1–3 min after stimulation was terminated (12.48±4.3% increase in arterial diameter at 2 min post stimulation, n = 21, Fig. 1B). Diameter and rCBF increase were dependent on the intensity and pulse width (Fig. 1C): Stimulation at 1 mA (200 µs) did not induce a significant increase in vascular diameter or rCBF (n = 7), however stimulation at 2 mA resulted in a significant increase in both vascular diameter and rCBF in 12 of 14 (86%) rats. Prolonging the pulse duration to 500 µs was associated with an additional significant increase in diameter and rCBF (Fig. 1C). No significant changes in vascular diameter or rCBF were observed in non-stimulated animals during 3 h of recordings (n = 6).

**Figure 2:**
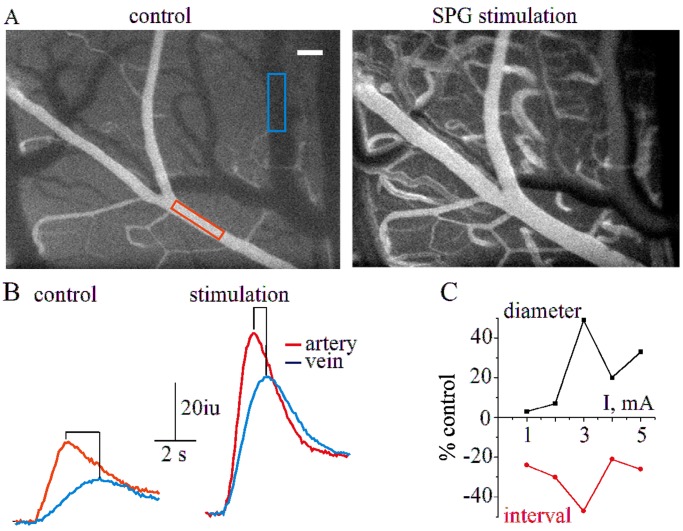
Fluorescent angiography during SPG stimulation A, Two representative angiographic images of cortical surface vessels under control conditions (left) and during SPG stimulation (3 mA, 500 µs, right, white bar represents 0.1 mm). B, Intensity curves (in arbitrary intensity units, iu) for the venous (blue) and arterial (red) compartments for the venous (blue) and arterial (red) compartments (marked in (A)) during control injection (left) and SPG stimulation (right). C, % change in diameter (black) and peak-to-peak (arterial-venous) interval (red) at different stimulation intensities (1–5 mA, 500 µs).

The effect of SPG stimulation on rCBF was also measured using fluorescent angiography (Fig. 2A). During SPG stimulation, intensity curves for the selected arteriole and venule showed a larger incline in signal intensity and a higher maximum, indicating increased blood flow (Fig. 2B). When the stimulation intensity was raised from 1 to 3 mA, the arterial diameter reached its maximum; a further increase in stimulation intensity led to a decrease in the vascular diameter (Fig. 2C). The arterial-venous peak-to-peak interval, an indirect measure of blood flow, generally decreased when the stimulation intensity was increased, suggesting that stimulation-induced vasodilatation is indeed associated with enhanced blood flow within the affected vasculature (Fig. 2B). In these experiments, stimulus-induced vasodilation and elevation of blood flow were not associated with a significant increase in BBB permeability measured using fluorescent angiography [20] (data not shown and see below).

**Figure 3:**
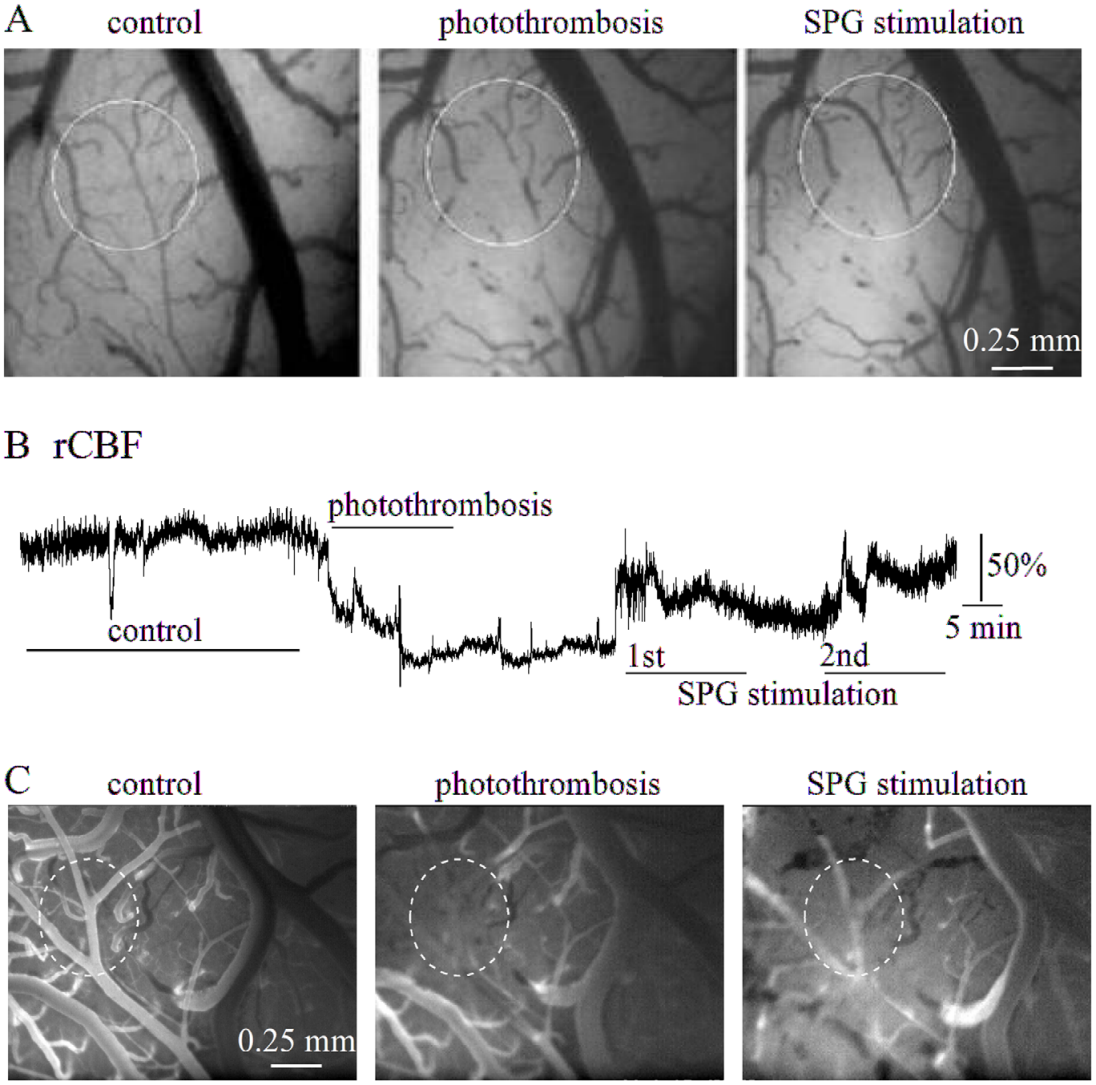
SPG stimulation in the RB-treated cortex. A, Brain surface images in a typical experiment before (left), and after (middle) photothrombosis and during SPG stimulation (2 mA, 500 µs, right). Note vasodilation and reperfusion of the thrombosed vessels (circled). B, Laser-Doppler recording in the same experiment as in (A), showing reduced rCBF during photothrombosis, which was partially reversible during SPG stimulation. C, Fluorescent angiography from a different rat before (left), after photothrombosis (middle) and during SPG stimulation (2 mA, 500 µs, right).

In the RB-treated cortex fluorescent angiography and laser-Doppler flowmetry confirmed the induction of intravascular thrombosis and the associated reduced blood flow within the injured vessels (Fig. 3A-B). In three of four RB-treated rats, SPG stimulation 5–15 min after the induction of photothrombosis induced partial recovery of rCBF, as measured using laser Doppler flowmetry. Notably, vasodilation of thrombosed vessels was evident, while fluorescent angiography confirmed partial reperfusion of the injured cortex (Fig. 3).

### SPG Stimulation in RB-Treated Animals Protects BBB Integrity and Reduces the Volume of Necrotic Cell Loss

**Figure 4:**
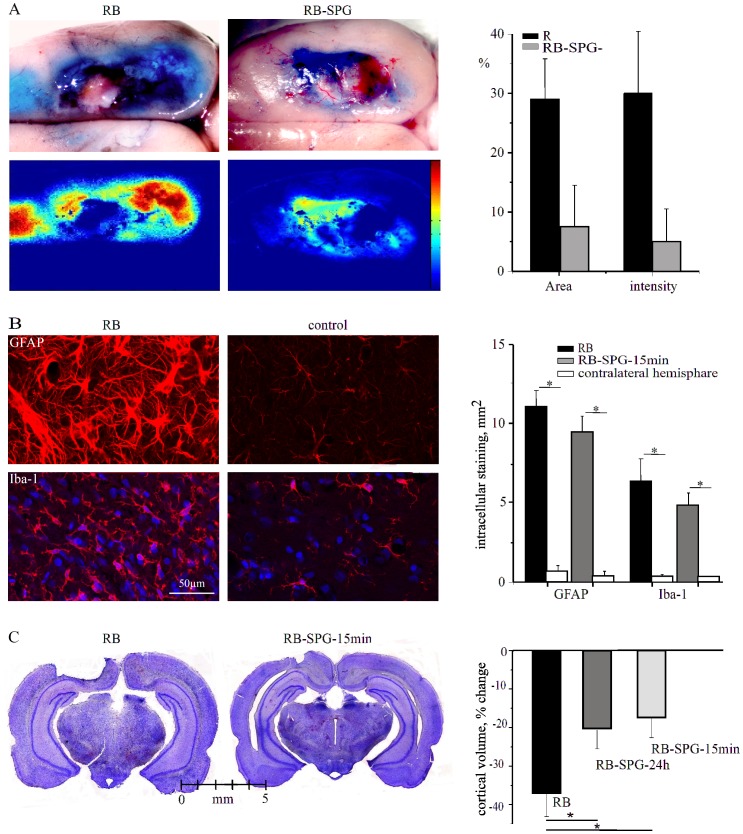
BBB breakdown, astroglial response and brain damage after SPG stimulation A, Two brain surface images after injection of Evans blue indicates BBB breakdown of RB-treated (left) and RB-SPG-15 min (right) rat brains. Images below display EB (blue color) intensity, color coded. Both the size of the area with increased EB and the intensity were decreased in SPG-treated rats compared to non-stimulated animals (right graph). B, Immunostaining against the astrocytic marker, GFAP (upper panels) and the microglial marker, Iba-1 (lower panel) in RB-treated (left) and the contra-lateral control hemisphere (right). Bar graphs show area measured with intracellular staining (see Methods). C, Coronal sections of brains from RB (left) and RB-SPG-15 min (right) animals. Bar graphs show change in cortical volume after photothrombosis. *p<0.05.

Vascular dysfunction with a leaky BBB is a hallmark of the peri-ischemic region [23]. We thus tested the effect of SPG stimulation on BBB opening in the RB model. Consistent with previous studies [24], a large region of the cortex surrounding the photothrombotic lesion showed intraparenchymal staining with an EB-albumin complex, indicating BBB dysfunction (Fig. 4A). In SPG-stimulated rats (n = 6), both the size of the stained cortical region and the intensity of the dye were smaller compared to those in RB-treated non-stimulated animals (n = 6, Fig. 4A). The peri-infarct zone was also associated with activation of astrocytes and microglia, as seen by enhanced immunostaining for GFAP and Iba-1, respectively (Fig. 4B). No differences in astrocytic or microglia stainings within the peri-infarct zone were found between SPG-stimulated and non-stimulated controls (Fig. 4B).

To measure the volume of brain loss following RB-induced ischemia, cortical volume was measured in the ipsilateral vs. the contralateral hemisphere two-three weeks after treatment (Fig. 4C, left). Importantly, rats stimulated either at 15 min or 24 h after photothrombosis showed a significant reduction in loss of brain tissue compared to non-treated controls (37.1±6.0; 20.6±4.8 and 17.9±5.1% reduction in cortical volume compared to the contralateral hemisphere for RB, n = 9; RB-SPG-15 min, n = 8 and RB-SPG-24 h, n = 6, respectively, Fig. 4C).

### SPG Stimulation in RB-Treated Animals Improves Cortical Functions

Since BBB dysfunction has been shown to lead to cortical dysfunction and seizures [25], [26], we used epidural electrodes placed above the peri-ischemic region to record ECoG network activity for six days after the induction of stroke (RB (n = 10), RB-SPG-15 min (n = 9), RB-SPG-24 h (n = 9)).

**Figure 5:**
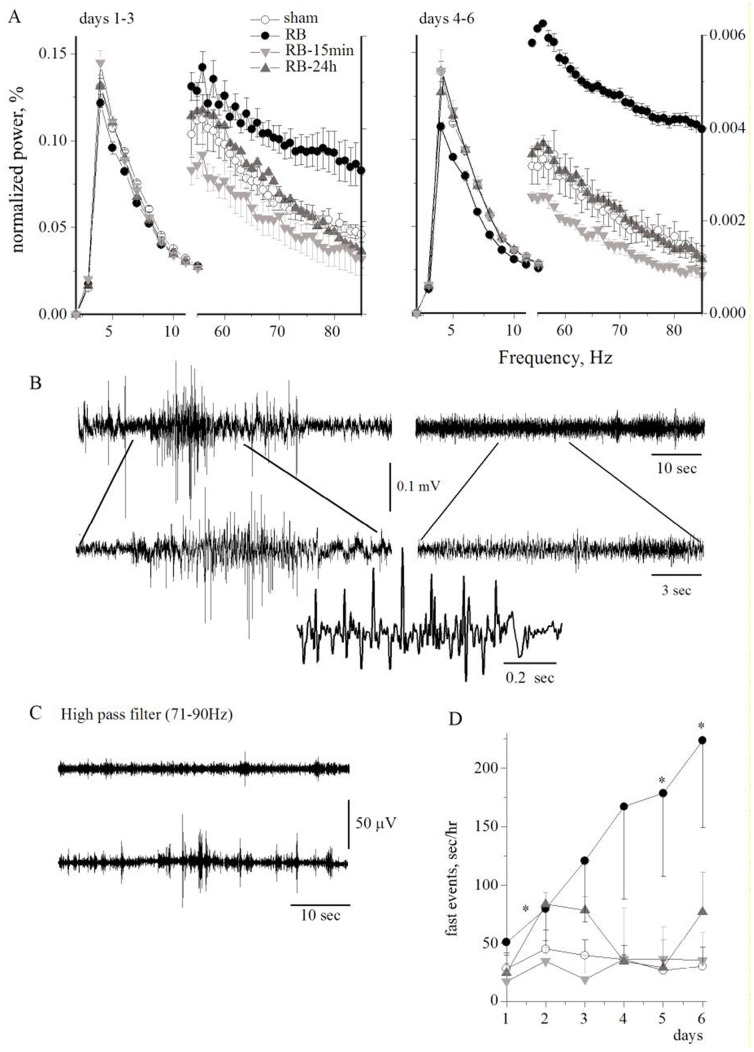
ECoG Fast Fourier Analysis: A, Averaged power of the normalized ECoG 1-3 (left) and 4–6 (right) days after photothrombosis in low and high frequency ranges (note the different right and left Y axis). B, Typical ECoG recordings of RB-treated (left) and sham animals (right). C, ECoG recording after high pass filter (71–90 Hz) of RB-treated (lower trace) and sham animals (upper trace). D, Duration of fast activity (sec/h) for the different treated groups. Symbols as in (A). *p<0.05.

Another group of animals (n = 8) were exposed to the same surgical procedures but without photothrombosis (sham). Differences in ECoG activity were measured using power spectrum analysis and compared between the groups (Fig. 5A). Normalized power in the low frequency range (<10 Hz) was decreased and in the high frequency range (>60 Hz) was increased over RB-treated cortices (Fig. 5A). Changes in power became significant from day 4 after photothrombosis and were most prominent in the 5–10 Hz and 46–90 Hz frequency bands. Importantly, SPG stimulation resulted in normalization of cortical activity, and both early (15 min)-treated and late (24 h)-treated groups showed ECoG activity similar to that of the sham group. The increased power in the high frequency range was due to the appearance of high amplitude, fast, seizure-like events in RB-treated animals but only rarely in controls (Fig. 5B). Indeed, the total time ECoG displayed high amplitude fast activity was significantly increased in the RB-treated group compared to sham controls or SPG-treated animals (Fig. 5C-D).

## Discussion

In the present study, we examined – for the first time– the effect of SPG stimulation on the diameter and blood flow in surface cortical vessels in healthy and ischemic brains. We further tested the effect of repetitive SPG stimulation on cortical electrophysiology within the peri-infarct region in the photothrombosis model. The main findings of this study were: (1) SPG stimulation led to intensity- and pulse width dependent vasodilation and increased rCBF; (2) SPG-induced vasodilation facilitated partial reperfusion of occluded vessels in the phothrombosis RB model; (3) the peri-ischemic zone showed increased permeability of the BBB, which was reduced in size both immediate and delayed stimulation of the SPG; (4) the necrotic core lesion was smaller following SPG treatment; (5) fast cortical electrical activity and seizure-like events were prominent from day 3 after photothrombosis and reduced to control levels in SPG treated animals.

This is the first study to show - using high-resolution direct imaging - a stimulation-induced significant and preferential vasodilation of cortical arterioles. Our findings confirm that in SPG stimulation, vasodilation is dependent on the stimulation intensity and pulse width (Fig. 1) within a narrow range of stimulation parameters (1–3 mA, 0.1–0.5 ms). It is of note that rCBF measurements by laser Doppler flowmeter or fluorescent angiography similarly showed 10–30% increase in the diameter of superficial arterioles and >100% increase in rCBF when stimulated at moderate-high (>1 mA) intensities. These results are consistent with previous studies showing 40–100% increase in rCBF using laser Doppler flowmeter [9], [11], mass spectrometry [27] or MRI perfusion [15], [28] and 10–40% increase in the diameter of the larger arterial brain suppliers in a dog model for sub-arachnoid hemorrhage [13]. A milder effect on rCBF was reported by Henninger and Fisher [15] in rats, when isoflurane anesthesia was used, perhaps because of the vasodilating effect of isoflurane [29]. While previous studies in monkeys demonstrated that SPG stimulation induces dilatation of constricted large vessels (internal carotid, anterior cerebral and middle cerebral arteries), in a sub-arachnoid hemorrhage model [30], our study shows that similar dilation occurs in small surface arterioles (but not venules) of the healthy brain. Recently, similar protocol has been shown to increase perfusion in MRI scans of rats following MCAO occlusion [28], suggesting that the vasodilation we observed in superficial vessels reflects a general response of both superficial and deep vessels to stimulation of the parasympathetic fibers of the greater superficial petrosal nerves [31].

**Figure 6:**
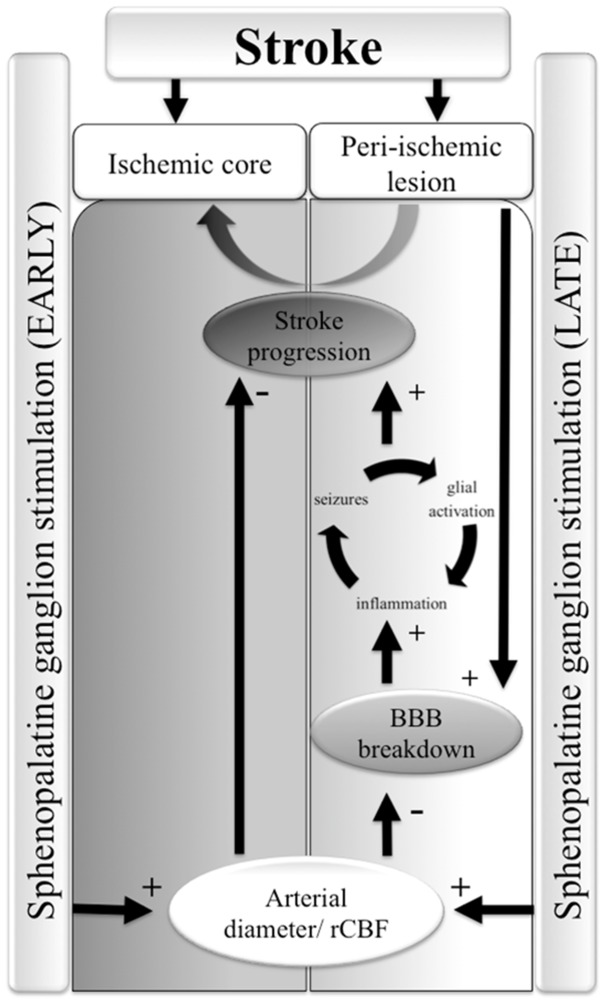
A working hypothesis on SPG-induced brain protection after stroke: The ischemic core is surrounded by a peri-ischemic region which is susceptible for an irreversible injury (AKA “stroke progression”). The peri-ischemic lesion is characterized by dysfunction of the blood-brain barrier (BBB) which induces neuronal hyper-excitability, spreading depolarizations and seizures, inflammation and cellular damage [4]. SPG stimulation at an early, post-insult therapeutic time window induces vasodilation and increased rCBF, sufficient to re-perfuse the ischemic core and to reduce lesion size. When stimulation is initiated at a delayed post-insult therapeutic window (24 h), vasodilation in the peri-ischemic lesion attenuates BBB injury and the associated neuronal hyper-excitability, thus preventing progression of the primary lesion.

Notably, using the direct imaging approach [20] we show for the first time in a stroke (photothrombosis) model, that SPG-induced vasodilation may result in immediate reperfusion of the ischemic core. Reperfusion was not associated with any apparent “steeling effect”, as indicated by laser Doppler recordings and angiography, documenting stimulation-mediated increase in perfusion of the peri-ischemic region as well (Fig. 3 and see below). Since SPG stimulation has been suggested to increase BBB permeability in the healthy and injured brain [13], and since stroke is often associated with BBB dysfunction, we evaluated the effect of stimulation on vascular permeability in the peri-ischemic brain. In contrast to previous studies [14], [32], SPG-induced stimulation protocols in the present study did not induce a significant increase in BBB permeability within the healthy brain. This could be attributed to the low intensity, short duration (<0.5 µs in our study vs. >1 ms in [32]), clinically tolerable stimulation applied, to the more focal stimulation method (bipolar stimulation in our study vs. monoplar stimulation in [32]) or to the avoidance of isoflurane anesthesia, which may contribute to BBB breakdown [33]. For the first time, we tested the delayed effect of SPG stimulation on vascular permeability in a rat stroke model. Remarkably, 24 h following photothrombosis, both the intensity and the extent of the area showing EB extravasation were significantly reduced in the SPG-treated group compered to RB group, indicating a reduction in BBB damage within the peri-ischemic region. The protective effect of SPG stimulation on BBB breakdown may be due to reperfusion of the ischemic core and reduced cellular damage and/or due to increased rCBF in the peri-ischemic brain itself. Since BBB breakdown induces a rapid inflammatory response [34] and activation of astrocytes [22], [35] we searched for differences in the immune-labeling of reactive microglia and astrocytes between the RB-treated groups. While no differences were found between the groups in the peri-ischemic region, we cannot exclude milder differences below the sensitivity of our detection methods, or functional differences in glial properties. Indeed, in consistent with recent studies demonstrating the role of BBB dysfunction and the associated astroglial response in the generation of seizures and delayed neuronal damage [4], [26], [36], [37], our ECoG recordings from the peri-ischemic, BBB dysfunctional cortex confirmed a gradual increase in the occurrence of seconds-long, fast and high amplitude, seizure-like events within the first week after photothrombosis. SPG stimulation and the subsequent milder BBB dysfunction were associated with normalization of brain activity and reduced occurrence of the seizure-like events. The decreased loss of brain tissue measured 2–3 weeks after treatment could result from the stimulus-induced vasodilation and increased oxygen supply to the ischemic core, but may also be directly related to the increased perfusion and decreased vascular (BBB) damage in the peri-ischemic brain. Future studies are required to reveal whether BBB dysfunction is directly related to stroke progression (due, for example, to the leakage of serum components into the brain tissue) or is simply a bystander biomarker for vascular pathology. Since ischemic damage in the core region is expected to become irreversible within few hours after the reduction of perfusion, it is tempting to speculate that the salvage of brain tissue in the 24 h post-insult stimulation protocol indicates that vascular damage around the ischemic core underlies stroke progression. This allows a beneficial effect for increased perfusion, even when initiated 24 h after the primary insult (Fig. 6). Accelerating BBB repair in the peri-ischemic region may also be beneficial in preventing delayed hemorrhagic transformation [38]. Furthermore, if this hypothesis is valid, BBB damage in the peri-ischemic brain may serve as a biomarker for predicting the efficacy of delayed treatment. The benefit of 24 h stimulation protocol is consistent with a recent study showing ameliorate MRI tissue characteristics in the rat MCAO model even when SPG has been started 18 h post-occlusion [28]. Treatment efficacy is emphasized in our study by the complete normalization of brain activity and the observed reduction of lesion size. Normalization of hyper-excitability in the peri-ischemic brain might be related to amelioration of blood flow and/or vessels’ integrity. While behavioral studies are awaited to confirm the functional and anatomical study performed here, the clinical implications of our results are emphasized by recent recordings in human patients following stroke or sub-arachnoid hemorrhage showing high frequency of seizures and spreading depolarizations within the peri-lesional brain and their potential role in delayed damage [39]–[42]. Together, these findings stress the potential of brain activity monitoring in stroke for diagnosis and treatment decisions and suggest a longer therapeutic window in selected patients.

In conclusion, this study shows that SPG-stimulation in rats, even 24 h after the induction of photothrombosis, improves cortical functions and reduces the extent of early BBB dysfunction as well as the size of the necrotic region. These results further strengthen the prevailing notion that SPG therapy may have a beneficial role in the treatment of stroke and emphasize potential mechanisms underlying the effects of SPG stimulation on peri-ischemic brain areas.
